# ER stress sensors at the ER-mitochondrial interface, controlling mitochondrial health in neurodegenerative diseases

**DOI:** 10.3389/fnins.2025.1665272

**Published:** 2025-12-15

**Authors:** Chaitanya Kunja, Vaishali Kumar, Pradeep Kodam, Chamundeshwari Devi Gopu, Shuvadeep Maity

**Affiliations:** Department of Biological Sciences, Birla Institute of Technology and Science (BITS)-Pilani (Hyderabad Campus), Hyderabad, Telangana, India

**Keywords:** ER-mitochondrial interactions, neurodegenerative diseases, ER stress sensors, mitochondrial health, IRE1, pERK, UPR signaling

## Abstract

The endoplasmic reticulum (ER) and mitochondria are essential organelles that interact closely at specialized sites known as ER-mitochondria-associated membranes (MAMs). MAM is enriched with proteins from both the ER and mitochondria. ER stress sensors—inositol-requiring enzyme 1 (IRE1) and protein kinase RNA-like ER kinase (PERK) — are traditionally recognized for their roles in the unfolded protein response (UPR), which mitigates proteotoxic stress. However, recent studies reveal their non-canonical functions at MAMs, where they regulate calcium signaling, mitochondrial dynamics, and apoptosis through interactions with MAM-resident proteins. Disruption of these pathways is implicated in various diseases, particularly neurodegenerative disorders. This review highlights the emerging roles of IRE1 and PERK in preserving mitochondrial function and their relevance to neurodegeneration. It also examines pharmacological strategies targeting these proteins, which influence both UPR signaling and ER-mitochondrial communication, offering a comprehensive perspective on their roles in health and disease.

## 1 Introduction

In eukaryotic cells, the endoplasmic reticulum (ER) is the largest membrane-bound organelle, forming an extensive network of interconnected tubules and sheets. Based on the presence of membrane-bound ribosomes, the ER has been classified into two distinct types: rough ER and smooth ER. Together, these structures support a wide array of essential cellular functions, including protein and lipid synthesis, processing, and trafficking (Vance, 1990; Jacquemyn et al., 2017; Rahhali et al., 2023). Importantly, in coordination with mitochondria, the ER plays a pivotal role in calcium (Ca^2+^) storage and release, as well as in organelle biogenesis. This ER-mitochondrial communication is facilitated through a dynamic region termed the mitochondria-associated ER membrane (MAM). MAMs are enriched with transmembrane proteins that coordinate diverse signaling pathways (Janikiewicz et al., 2018; Missiroli et al., 2018). As a central hub for the synthesis of membrane and secretory proteins, the ER is particularly vulnerable to disruptions in protein folding, which may arise from environmental or pathological stressors. The accumulation of misfolded proteins triggers a condition known as ER stress, which activates a conserved signaling cascade termed the unfolded protein response of the ER (UPR^ER^). ER transmembrane proteins, Inositol-requiring enzyme 1 (IRE1), protein kinase RNA-like ER kinase (PERK), and Activating transcription factor 6 (ATF6) serve as primary sensors of ER stress and initiate the UPR^ER^. Protein misfolding conditions can also activate mitochondrial UPR (UPR^mt^) primarily through activation of three transcription factors activating transcription factor 5 (ATF5), activating transcription factor 4 (ATF4), and C/EBP homologous protein (CHOP), which play crucial roles in maintaining mitochondrial health.

Interestingly, several studies have revealed that IRE1 and PERK are frequently localized at MAM, performing functions beyond their canonical roles in stress sensing (Verfaillie et al., 2012; Carreras-Sureda et al., 2019). In this review, we have discussed the non-canonical roles of IRE1 and PERK contributing towards different physiological processes other than UPR^ER^. Further, we have described their implications in the pathogenesis of neurodegenerative diseases and reviewed therapeutic strategies targeting these proteins.

## 2 ER stress sensors at mitochondria-associated membranes (MAMs)

Mitochondria-associated membranes (MAMs) are specialized contact sites where the ER and mitochondria are closely tethered—within a distance of approximately 10–25 nm—by a set of bridging proteins, without complete membrane fusion. Technological advancements and accumulating evidence have revealed that MAMs function as intricate signaling hubs, composed of proteins derived from both the ER and the outer mitochondrial membrane (Csordás et al., 2006). During altered physiological conditions like ER and oxidative stress, the communication between the ER and mitochondria becomes vital for adapting to rapidly changing bioenergetic demands (Carreras-Sureda et al., 2019; Bassot et al., 2023). IRE1 and PERK regulate different signaling pathways in both canonical and non-canonical ways.

### 2.1 ER stress sensors activate their canonical signaling unfolded protein response (UPR) during ER stress

**Figure 1:**
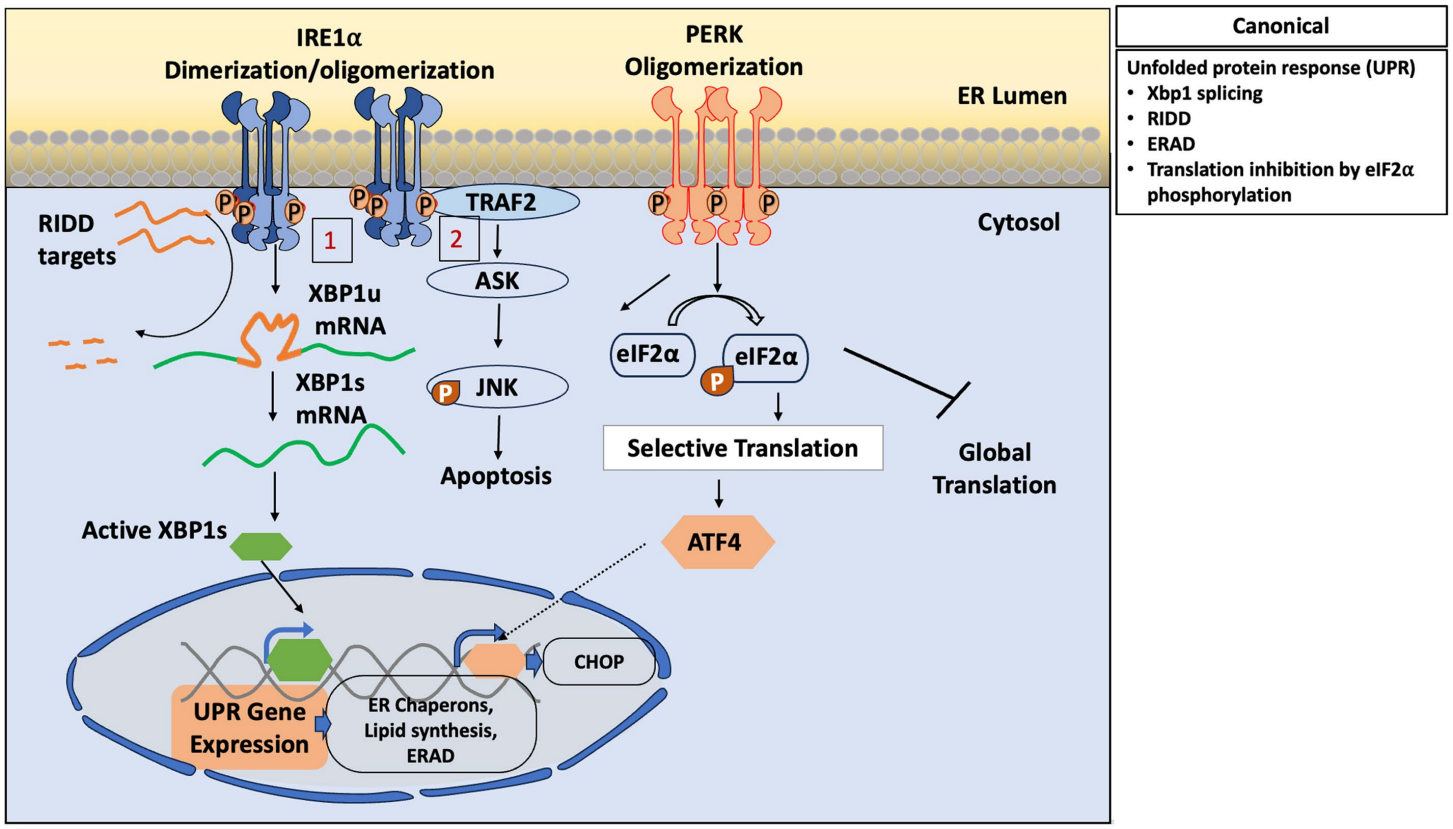
Canonical pathway of ER stress activated by IRE1a and PERK in mammalian cells.: IRE1a undergoes dimerization oligomerization and autophosphorylation, leading to two main downstream events: (1) Unconventional splicing of XBP1u (unspliced) mRNA to produce spliced XBP1(XBP1s), which acts as an active transcription factor controlling UPR target gene expression [ER chaperons, Lipid biosynthesis, ER- associated degradation (ERAD)]. (2) interaction with TRAF2 to activate the JNK pathway mediated apoptosis via ASK1. PERK oligomerizes and auto phosphorylates, resulting in phosphorylation of elF2a, which inhibits global protein translation, allowing selective translation of ATF4. ATF4 functions as a transcription factor to induce genes involved in protein folding, and CHOP mediated apoptosis in case of prolonged stress.

Typically, Inositol-requiring enzyme 1 (IRE1), encoded by the *Ern1* gene (Endoplasmic Reticulum to Nucleus Signaling 1), is one of the most evolutionarily conserved transmembrane proteins of the ER and serves as a critical sensor of ER stress (Mori et al., 1993) (Figure 1). The canonical role of IRE1 was first identified in yeast through genetic screening, where it was recognized as a core component of the unfolded protein response (UPR) (Cox et al., 1993). In mammals, IRE1 exists in two isoforms: IRE1α and IRE1β. While budding yeast harbours only a single isoform, IRE1α is the predominant and ubiquitously expressed form in mammalian cells (Tirasophon et al., 1998). During its canonical response, IRE1α undergoes homodimerization after sensing misfolded protein accumulation inside the ER. Dimerization followed by autophosphorylation and subsequent conformational change activates its cytosolic RNase domain, which catalyses the unconventional splicing of *HAC1* (in yeast) or *XBP1* (in mammals) mRNA (Ron and Walter, 2007). The spliced form of *XBP1* (or *HAC1* in yeast) encodes a transcription factor that upregulates the transcription of UPR genes involved in protein folding, ER-associated degradation (ERAD), and lipid biosynthesis. In addition to these transcriptional responses, IRE1 also mediates regulated IRE1-dependent decay (RIDD) of select mRNAs, thereby reducing the protein folding burden on the ER and contributing to the restoration of proteostasis (Hollien and Weissman, 2006; Tam et al., 2014). Active IRE1α binds the adaptor protein TNFR-associated factor 2 (TRAF2), triggering the activation of the apoptosis signal-regulating kinase 1 (ASK1) and c-Jun/N-terminal kinase (JNK) pathway (Urano et al., 2000; Nishitoh et al., 2002).

PERK signaling is initiated by the phosphorylation at Ser51 of the α subunit of eukaryotic translation initiation factor 2 (eIF2α), resulting in a temporary cessation of global protein synthesis in response to ER stress or integrated stress response (ISR) (Figure 1). Interestingly, despite the overall inhibition of translation, a selective subset of translation remains active through stress-responsive transcription factors like ATF4, allowing the continued transcription of genes that regulate autophagy, ERAD, cell viability, and apoptosis (Almeida et al., 2022). ATF4 acts as a transcription factor of two key target genes, Growth Arrest and DNA damage 34 (GADD34) and C/EBP Homologous Protein (CHOP). GADD34, along with protein phosphatase 1 (PP1), dephosphorylates eukaryotic initiation factor 2α (eIF2α) to release the global translation arrest (Brush et al., 2003). On the other hand, CHOP can act as a rheostat that helps decide cellular fate (proliferation or apoptosis) during ER stress (Liu et al., 2024). CHOP can also control mitochondrial health as part of stress adaptation (Kaspar et al., 2021). The overlapping activation of PERK either via ER stress or ISR, can promote remodeling of mitochondria through both transcriptional as well as translational signaling (Almeida et al., 2022).

Beyond their canonical roles in detecting misfolded proteins and initiating the UPR, these proteins are thus increasingly recognized for their involvement in broader physiological functions mediated through ER-mitochondrial interactions (Figure 1). Here, we have focused on the ER-mitochondrial interactions governed by IRE1/PERK and other proteins to maintain cellular energy balance, redox status, and overall metabolic function.

### 2.2 IRE1—arm controlling ER-mitochondrial communication

**Figure 2:**
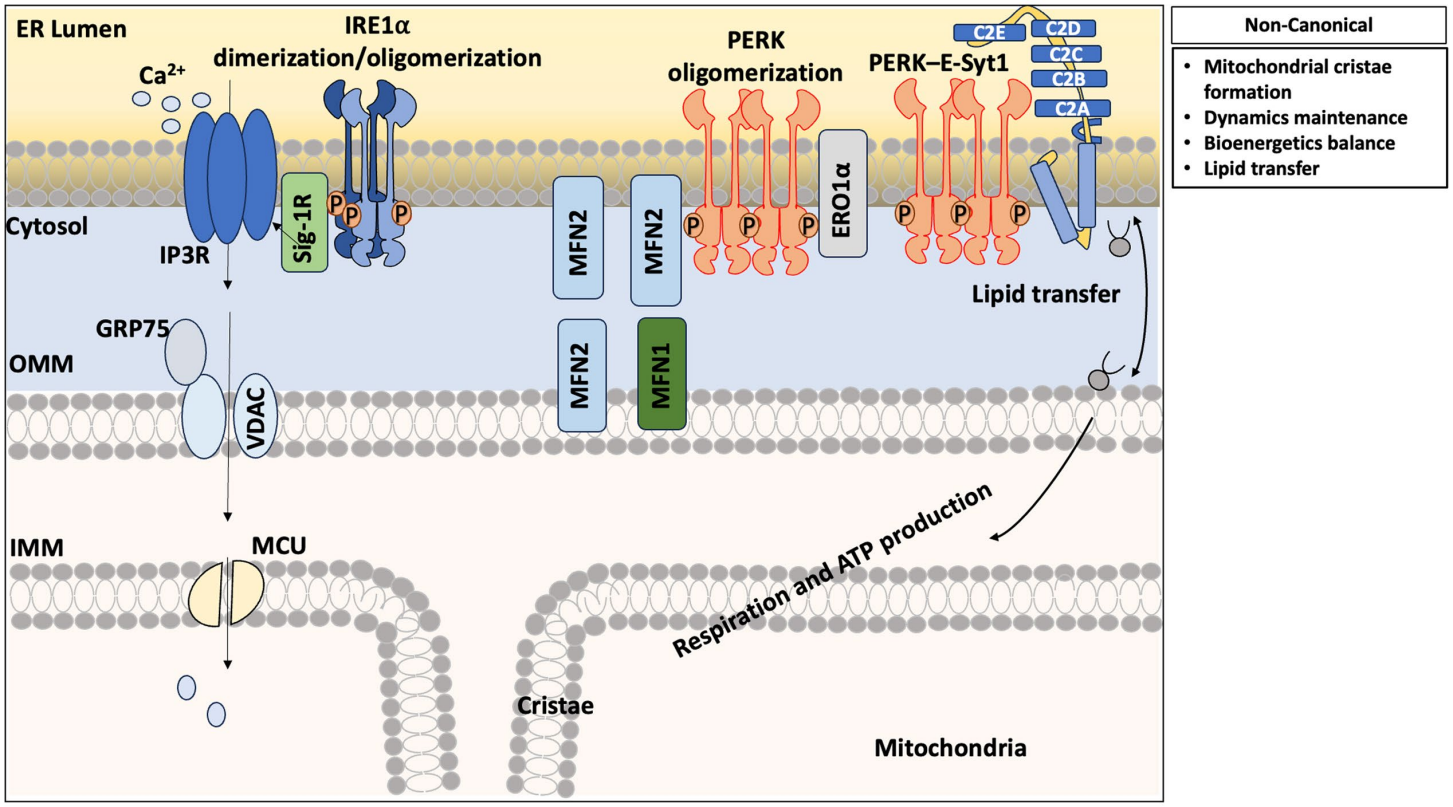
Non-canonical pathway activated by IRE1a and PERK: At MAM, IRE1a interacts with ion channel Sigma-1 receptor (Sig-1R) and inositol triphosphate receptor (IP3R), facilitating calcium transfer from the ER to mitochondria through VDAC-GRP75 complex, supporting mitochondrial respiration and ATP production. interaction of PERK with MFN1 and MFN2 contributes to ER-mitochondrial tethering and maintenance of morphology. The PERK-E-Syt1 complex involves in lipid transfer across membranes. Overall these interactions show non-canonical role of UPR sensors in the regulation of mitochondrial cristae formation, maintenance of dynamics, bioenergetic balance, and lipid homeostasis.

In addition to its canonical function in the UPR, IRE1 plays a critical role in regulating different physiological processes, including ER-mitochondrial communication through its localization at MAM in various cellular contexts (Figure 2; Table 1).

**Table 1:** Non-canonical role of ER stress sensors IRE1 and PERK on organellar health.

ER stress sensors/signaling pathway and a component of MAM	Organism	Interacting protein	Function	Reference
IRE1	*Saccharomyces cerevisiae*	—	Diauxic shift induces splicing of the HAC1 mRNA (IRE1-HAC1 axis) Activation of Ire1 is mediated by reactive oxygen species (ROS) but not accumulation of unfolded proteins	Tran et al. (2019)
IRE1	*Saccharomyces cerevisiae*	—	Deletion correlates with reduction of mitochondrial HSP60 expression	Maity et al. (2014)
IRE1α / (also known as ERN1)	MCF-7 CHO cells	Sigma 1R (SIGMAR1)	Stabilizes IP3R. Prolongs Ca2 + release. Mediates stabilization of IRE1 and sensitization to mitochondria-derived ROS	Hayashi and Su (2007) and Mori et al. (2013)
IRE1α	HeLa, MEF, IRE1α conditional KO mice	IP_3_R (ER)	Scaffold function. Regulates the distribution of IP_3_R at MAMs. Promotes enhanced mitochondrial Ca2 + uptake	Carreras-Sureda et al. (2019)
IRE1αa	HEK293, MCF-7, IRE1α conditional KO mice	—	IRE1α sustains the mitochondrial oxygen consumption rate	Navarro-Betancourt et al. (2020)
IRE1α-XBP1	SH-SY5Y Cells	MITOL	IRE1α-XBP1 Affects the Mitochondrial Function of Aβ25–35-Treated SH-SY5Y Cells by Regulating Mitochondria-Associated Endoplasmic Reticulum Membranes	Chu et al. (2021)
IRE1-XBP1 axis	SH-SY5Y Cells	Increases interaction of MAM proteins (IP3R-Grp75 and VDAC1)	restoring the mitochondrial function and reducing the interaction at the MAM region	Chu et al. (2021)
IRE1-XBP1 axis	IRE1/XBP KO Mice		The IRE1α-XBP1 axis supports the glycolytic switch in response to inflammatory stimuli	English et al. (2023b)
IRE1-XBP1 axis	*C. elegans*, PC12,	—	The XBP1s axis increases HSPA5/BiP, degradation of lipid droplets	Daniele et al. (2018)
PERK				
PERK (EIF2AK3)/Pek-1/PEK1	Mammals/*C elegans*/*Drosophila*	ESYT1 (ER), Ero1a (ER)	Depletion disrupts Mitochondria-ER contact sites (MERCs) (in MEFs). Regulates Ca2 + signaling, lipid transfer, apoptosis induction, mitochondrial dynamics, and MERC protein oxidation	Verfaillie et al. (2012), Bassot et al. (2023), and Sassano et al. (2023)
PERK- YME1L	HeLa, MEF, HEK293T	YME1L (mitochondrial inner membrane protease)	ER stress-dependent increases in both cellular phosphatidic acid (PA) and YME1L-dependent degradation of the mitochondrial PA transporter, PRELID1	Lebeau et al. (2018) and Perea et al. (2023)
PERK—GA-binding protein transcription factor α subunit (GABPα) signaling axis	HEK293, ICR mice (SLC),	PERK autophosphorylation activate GABPα during BA differentiation	GABPα Mediated activation of mitochondrial inner membrane protein biogenesis during BA differentiation	Kato et al. (2020)
cGAS–STING–PERK pathway	HEK293, HeLa, HCT116, DLD1, MEF and C57BL/6 mice	STING-cGAMP (ER)	STING-cGAMP binds with PERK, directly activating its intracellular domains, which precedes TBK1–IRF3 activation. Impact over Innate immunity control, senescence and organ fibrosis	Zhang et al. (2022)
PERK-OGT-TOM70 signaling axis	BAT1, HEK293T and C57BL/6 mice	TOM70 glycosylation (mitochondria)	PERK-activated OGT glycosylates specific substrates including TOM70 to promote mitochondrial MIC19 import efficiency and cristae formation	Latorre-Muro et al. (2021)

Mori et al., 2013 reported that IRE1α is highly enriched at MAM in Chinese hamster ovary (CHO) cells. In this study, the authors identified the Sigma-1 receptor (Sig-1R), a calcium-sensitive and ligand-regulated chaperone, as a crucial component of this interface. Under normal physiological conditions, Sig-1R forms a complex with the ER chaperone BiP. However, Sig-1R dissociates from BiP upon ER calcium depletion and stabilizes inositol 1,4,5-trisphosphate receptors (IP3Rs), facilitating sustained calcium signaling from the ER to mitochondria. This was the first demonstration of an ER-resident chaperone directly modulating mitochondrial calcium signaling. Importantly, another study found Sig-1R also interacts physically with IRE1α at MAMs under ER stress conditions. This interaction is reactive oxygen species (ROS)-dependent and contributes to the stabilization of IRE1α at the MAM, thereby prolonging the IRE1-XBP1 signaling axis and promoting cell survival during stress (Mori et al., 2013).

Further insights into the regulatory role of IRE1α were provided by Takeda et al. (2019), who demonstrated that mitochondrial ubiquitin ligase MITOL (also known as MARCH 5) prevents ER stress-induced apoptosis by ubiquitylating IRE1α at MAM. MITOL-mediated K63-linked chain ubiquitination of IRE1α suppresses IRE1α hyper-oligomerization, thereby limiting its pro-apoptotic signaling and reducing regulated IRE1-dependent decay (RIDD) during ER stress (Takeda et al., 2019). Here, MAM serves as a signaling site for this protective adaptation, helping fine-tune IRE1α’s activity under stress conditions.

Expanding on the non-canonical functions of IRE1α, Carreras-Sureda et al. reported that conditional knockout of IRE1α in mouse liver resulted in significant alteration of mitochondrial metabolism. Interestingly, the study pointed out a reduction of MAM proteins but not mitochondrial proteins. *In vitro* studies with combinations of different mutants of IRE1α further revealed that a direct interaction between IRE1α and IP3R1 is essential for mitochondrial calcium uptake (Carreras-Sureda et al., 2019). These findings highlight the physiological importance of IRE1α in maintaining ER-mitochondrial homeostasis beyond its classical role in the UPR.

The role of IRE1α in maintaining mitochondrial integrity has also been demonstrated in podocytes or Glomerular epithelial cells (GEC). Navarro-Betancourt *et al.* showed that podocyte-specific knockout of IRE1α in mice led to significant mitochondrial dysfunction. These cells exhibited reduced respiratory capacity, diminished mitochondrial membrane potential, and structural abnormalities. Electron microscopy revealed loss of cristae, increased mitochondrial roundness, and decreased matrix density—hallmarks of impaired mitochondrial health. These findings underscore the essential role of IRE1α in preserving mitochondrial structure and function, particularly in metabolically active cells (Navarro-Betancourt et al., 2020).

The IRE1α–XBP1 axis has also been shown to regulate metabolic flexibility in immune cells. In macrophages, activation of this pathway is essential for the metabolic shift toward glycolysis following lipopolysaccharide (LPS) infection. This glycolytic switch enables rapid ATP production, which is critical for sustaining the pro-inflammatory response. Conditional knockout of IRE1α or XBP1 in macrophages impaired the expression of glycolytic genes, disrupted glycolytic flux, and compromised mitochondrial metabolism. As a result, energy production was insufficient to maintain immune activation, highlighting the axis’s role in coordinating energy metabolism during immune stress (English et al., 2023a).

In the neuronal system, the IRE1α-XBP1 signaling axis plays a pivotal role at MAMs, where it helps maintain ER-mitochondrial tethering and supports mitochondrial function. A recent study demonstrated that treatment of SH-SY5Y neuroblastoma cells with Aβ₍₂₅_−_₃₅₎ activates the IRE1–XBP1 pathway, enhancing interactions with key MAM proteins such as IP3R, GRP75, and VDAC1. This leads to increased calcium transfer from the ER to mitochondria. However, excessive calcium influx can result in mitochondrial dysfunction. Notably, inhibition of the IRE1–XBP1 axis restored mitochondrial function and reduced MAM-associated interactions, suggesting that this pathway fine-tunes ER-mitochondrial communication to preserve mitochondrial health under stress conditions (Chu et al., 2021).

IRE1 which typically activates pro-survival signaling pathways can induce apoptosis for prolonged ER stress. Due to its ability to be involved in two opposing functions, the activation of inhibition of this pathway leads to different effects based on the physiological condition of the cells. For example, chronic ER stress has been associated with retinal degeneration and diabetes. Thus, small molecule’s targeted inhibition of IRE1α was protective against retinal degeneration in the rat model and against pancreatic *β*-cells in the Akita diabetic mouse model (Ghosh et al., 2014). Interestingly, not the ER stress pathways but IRE1*α* knockdown has been shown to induce cell death in SH-SY5Y by disrupting intracellular Ca2 + homeostasis (Son et al., 2014). The RIDD via UPR sensor IRE1α transiently catalyzed *DR5* mRNA decay. This allows time for adaptation during chronic ER stress, promoting survival by inhibiting pro-apoptotic proteins like DR5 (Lu et al., 2014). Using Drosophila model of PD Yan et al. found that IRE1 promotes neuronal death of dopaminergic neurons and locomotor impairment through activating autophagic pathways. Thus, inhibiting IRE1 or its downstream autophagic proteins (ATG7) ameliorated the progression of α-synuclein-caused Parkinson’s disease (Yan et al., 2019; Hetz et al., 2020; Kim et al., 2022). On the contrary, genetic disruption of the ER stress sensor IRE1 accelerated age-related cognitive decline. In the context of brain aging, a mouse brain study demonstrated that impairment of UPR signaling begins with age. Over-expression XBP1s or XBP1s gene delivery to aged mice not only improved age associated phenotypes but also reverted brain dysfunction, indicating sustained expression of IRE1α-XBP1 pathway is essential to protect against age and age related disorders (Cabral-Miranda et al., 2022a). Further, the involvement of the IRE1-PERK arm as a part of MAM in controlling neurological disorders is discussed in a separate section. Collectively, these findings establish the IRE1–XBP1 axis as a key regulator of cellular survival, which also coordinates with other pathways, including mitochondrial function, depending on various cellular contexts.

### 2.3 PERK—arm controlling ER-mitochondrial communication

PERK maintains mitochondrial homeostasis, mitochondrial cristae junctions, and bioenergetics depending on the needs of cells. During ER stress, a discordant behavior of transcription and translation was observed by Rendleman et al. (2018). An in-depth integrated multi-omics study pointed out that even during the halt of the global translation, a subset of genes supports the rerouting of energy production via upregulated translation, but not transcription, of Complexes I-IV from the oxidative phosphorylation pathway. At the same time, Lebeau et al. (2018) pointed out stress-induced hyperfusion of mitochondria through PERK-mediated signaling in coordination with the inner membrane protease YME1L. The use of IRE1, PKA, HDAC, and PERK inhibitors revealed that YME1L supports mitochondrial elongation via PERK signaling during ER stress. A follow-up study showed that PERK regulates phosphatidic acid (PA) levels at the outer mitochondrial membrane by promoting YME1L-dependent degradation of the PA transporter PRELID. This mechanism facilitates mitochondrial fusion, highlighting PERK’s critical role in mitochondrial dynamics under stress (Perea et al., 2023). Contemporary research has shed light on the metabolic adaptation of mitochondria during early ER stress via ERO1α. At the early phase of ER stress, PERK interacts with oxidoreductase ERO1α, followed by the formation of a heterocomplex (PERK-ERO1⍺) through the C-terminal active site of ERO1⍺ and cysteine 216 of PERK. This redox-dependent complex with ERO1α promotes MAM protein oxidation and enhances ER-mitochondrial Ca^2+^ flux. An earlier interactome analysis revealed that PERK aids Ca^2+^ signaling by interacting with the SERCA pump (Sassano et al., 2021). These interactions help to maintain mitochondrial dynamics and bioenergetic balance while limiting oxidative stress (Bassot et al., 2023). PERK deficient cells showed altered vulnerability during ROS-mediated ER stress following ROS signaling between the ER and mitochondria (Verfaillie et al., 2012).

PERK interacts with the actin-binding protein Filamin A (FLNA), which plays a key role in F-actin remodelling. This PERK–FLNA axis is essential for expanding ER–plasma membrane (PM) contacts following ER Ca^2+^ depletion. Loss of PERK disrupts F-actin organization, alters the F/G actin ratio, and reduces cell motility. Notably, this activity is driven by Ca^2+^ signaling and occurs independently of classical UPR pathways. These findings further underscore PERK’s critical role in regulating mitochondrial dynamics and cytoskeletal integrity (Muñoz et al., 2013). PERK also plays a key role as a regulator of lipid trafficking at ER-mitochondria contact sites through its interaction with the lipid transfer protein E-Syt1. Disruption of this PERK–E-Syt1 complex impairs lipid transfer and compromises mitochondrial respiratory capacity (Sassano et al., 2023). During cold stress or high energy demand, PERK facilitates mitochondrial cristae formation and respiration by activating O-linked N-acetylglucosamine transferase (OGT), which glycosylates TOM70 at Ser94 and enhances MIC19 import in mitochondria (Latorre-Muro et al., 2021).

Fusion proteins, Mitofusin 1 (MFN1) and Mitofusin 2 (MFN2), are found to interact with PERK. Mfn2 can act as an upstream modulator of PERK. Ablation of Mfn2 causes sustained kinase activation of PERK. In a healthy condition, Mfn2 keeps PERK in an inactive state. An altered environment, such as a high glucose level, causes activation of PERK after a reduction in the Mfn2 level and a disturbance of MAM signaling with subsequent mitochondrial dysfunction (Muñoz et al., 2013).

Mitochondrial thermogenesis in brown adipose tissue is also regulated by the ER-resident UPR sensor PERK. During brown adipocyte differentiation, PERK becomes phosphorylated independently of ER stress. This activation enhances the transcriptional activity of GABPα, which is essential for mitochondrial inner membrane protein biogenesis. Although GABPα protein expression remains unchanged in PERK-deficient cells, its transcriptional activity is significantly reduced—an effect not observed in IRE1α- or ATF6-deficient cells—highlighting a unique PERK–GABPα signaling axis that may be targeted to boost energy metabolism without disrupting ER homeostasis (Kato et al., 2020).

Beyond controlling mitochondria, PERK plays a critical role in inflammation. The nucleic acid sensor cGAS detects cytosolic DNA from invading pathogens and synthesizes the secondary messenger 2′3′-cGAMP. This molecule binds to the ER-resident adaptor protein STING, a key player in innate immunity. STING engages PERK via its intracellular domain upon activation, triggering non-canonical activation of PERK. Activated PERK then phosphorylates eIF2α, initiating translational programs that support inflammatory and survival responses (Zhang et al., 2024).

## 3 IRE1/PERK at the interface of neurodegenerative disease

Neurodegenerative diseases, characterized by the progressive dysfunction and loss of neurons, are increasingly linked to disruptions of cellular proteostasis, particularly in the ER (Tam et al., 2014). As mentioned earlier, PERK, plays a pivotal role in maintaining cellular homeostasis under ER stress conditions (Shacham et al., 2021). However, under chronic ER stress, sustained PERK activation can shift from protective to deleterious, contributing to neuronal dysfunction and death (Xu et al., 2017). Increased levels of phosphorylated PERK (p-PERK) and phosphorylated eIF2α (p-eIF2α) were found in the brains of patients with neurodegenerative diseases, suggesting that ER stress and PERK activation may play a role in disease pathogenesis (Bell et al., 2016). Indeed, PERK activation has been directly linked to synaptic dysfunction, impaired long-term potentiation, and cognitive deficits. A significant example of PERK’s role in neurodegeneration comes from a study on tauopathies — a class of neurodegenerative diseases marked by tau aggregation. Park et al. elucidated that PERK (EIF2AK3) helps in preventing tau aggregation. Using HEK293 cells expressing TauRD (P301S)-YFP and PERK+/+ and PERK−/− mouse embryonic fibroblasts, authors demonstrated that PERK activation mitigates tau aggregation, while its inhibition exacerbates pathology. Studies on tauopathy patient brain tissue exhibited a correlation between diminished PERK signaling and increased tau pathology, underscoring the protective role of PERK against tau aggregation and suggesting a potential therapeutic target, particularly in individuals with tauopathy-associated PERK hypomorph alleles (Park et al., 2023). Conversely, downregulation of PERK was found to be beneficial for neurodegenerative conditions. Wang et al. demonstrated that PERK downregulation protects rat Müller glial cells under oxygen–glucose deprivation (OGD) (Wang et al., 2023). Furthermore, OGD-induced phosphorylated tau (p-Tau) expression in rMC-1 cells was diminished by PERK knockout, implicating PERK’s role in tau-related pathology under stress. PERK knockout reduced the expression of autophagy-related proteins (LC3 and Beclin1) and apoptotic markers, improving cellular viability. PERK can reduce tau phosphorylation and autophagic stress, offering protective benefits to retinal neurons and glia (Chiu et al., 2023). Interestingly, a recent study found that the selective removal of astrocytic PERK decreases toxic aggregation of *β*-amyloid and tau proteins with enhanced glymphatic clearance and improved cognitive performance in mice (Chen et al., 2025). In Alzheimer’s disease (AD), IRE1 activation has been correlated with increased amyloid-beta (Aβ) production and tau phosphorylation, thereby exacerbating neurodegenerative processes. Notably, activation of the IRE1α/TRAF2/JNK signaling pathway has been implicated in neuronal apoptosis across several neurodegenerative disorders (Wan et al., 2020). Targeting PERK and IRE1 presents a promising therapeutic strategy given their wide-reaching impact. Genetic or pharmacological modulation of IRE1 can significantly affect cell viability (Siwecka et al., 2021). Ablation of IRE1’s RNase domain reduces amyloid deposits, Aβ oligomer levels, and astrocyte activation in the brain of a mouse model (Duran-Aniotz et al., 2017). A very recent study identified a novel non-canonical function of the IRE1α–Xbp1 branch in regulating the proteostasis of poly (GR) via preventing its accumulation in Amyotrophic Lateral Sclerosis (ALS). Using different cellular and *in vivo* models authors demonstrated that ectopic expression of IRE1 or its downstream effector XBP1s or pharmacological activation of IRE1 significantly reduced poly (GR) protein levels and ameliorated associated proteotoxic phenotype (Li et al., 2025a).

Huntington’s disease (HD) is another condition where sustained UPR activation contributes to disease pathology. Mutant huntingtin protein (mHTT) leads to chronic ER stress in HD models, with upregulation of markers such as GRP78 and CHOP observed in the hippocampus even at early disease stages. In R6/1 HD mice, ER stress appears to be mediated by PERK hyperactivation, which impairs cognition through prolonged suppression of essential protein synthesis for plasticity and memory. Notably, genetic reduction of GRP78 expression mitigates spatial and recognition memory deficits, suggesting targeted therapeutic intervention can be achieved by modulating these pathways (Shacham et al., 2021; Espina et al., 2023).

Further evidence of the protective role of IRE1 comes from studies involving parkin overexpression. Both *in vitro* and *in vivo* studies demonstrated that increased XBP1s levels through overexpression of parkin potentially confer resistance to PD-related toxins in dopaminergic neurons. In PD, cell survival has been shown to be linked with nitric oxide (NO) exposure. Studies using parkin and its pathogenic mutants in SH-SY5Y neuroblastoma cells identified the protective role of the IRE1α/XBP1 signaling axis against NO-induced apoptosis. Parkin also enhances the activity of the RNase domain of IRE1α following treatment with nitric oxide (NO) donor, S-nitroso-N-acetyl penicillamine (SNAP). The protective activity of parkin was found to be compromised after pharmacological inhibition or genetic knockdown of the RNase activity. Pathogenic parkin mutants showed a lower ability to activate the IRE1α/XBP1 signaling (Chiu et al., 2023). In Marinesco-Sjögren syndrome (MSS), PERK inhibition delays neurodegeneration and improves motor function (Grande et al., 2018).

Beyond neurodegenerative diseases, multiple studies have consistently highlighted the connection between ER stress. ER stress markers are also dysregulated in neuropsychiatric disorders. For instance, Kim et al. and Xue et al., demonstrated altered expression of BiP, PERK, and XBP1 in the prefrontal cortex, along with elevated serum levels of ATF6 and XBP1 in individuals with schizophrenia. These molecular signatures suggest that ATF6 and XBP1 may serve as biomarkers and functional contributors to schizophrenia. Similarly, individuals with bipolar disorder (BD) show an impaired UPR under ER stress. A specific SNP (–116C → G) in the XBP1 promoter has been associated with increased BD risk, while lymphoblasts from BD patients exhibit reduced expression of UPR genes such as XBP1 and CHOP under ER stress conditions (Kim et al., 2021; Xue et al., 2023). In the pathophysiology of Major Depressive Disorder (MDD), elevated levels of ER stress markers such as GRP78, CHOP, and XBP1 have been found in both animal models and human subjects with depression. Notably, activation of the PERK-eIF2α pathway in the hippocampus is associated with decreased brain-derived neurotrophic factor (BDNF) levels and depression-like behavior. Interestingly, inhibiting PERK improves memory performance, emphasizing its role in cognition (Sharma et al., 2018). Elevated expression of ER stress-related genes has also been reported in learned helplessness models and post-mortem brain tissue from MDD patients, especially those who died by suicide. Furthermore, peripheral blood cells of MDD patients exhibit heightened ER stress markers, further affirming the role of ER stress in MDD pathophysiology (Nevell et al., 2014; Behnke et al., 2016).

The role of UPR extends beyond pathology to developmental processes. During early brain development, UPR activation, particularly via IRE1 and PERK pathways, significantly influences neurogenesis. Mouse embryonic stem cell models have shown that UPR induction promotes neuronal over glial differentiation, underscoring its impact on lineage specification. The PERK/ATF4 signaling axis is critical for neurogenesis and proper neuron positioning in the developing cortex (Laguesse et al., 2015). However, excessive ER stress during cortical development, such as that induced by reduced codon translation rates, may skew neurogenesis toward direct pathways, potentially leading to microcephaly.

UPR elements also regulate neurogenesis and synaptic connectivity. Specifically, BDNF-induced UPR signaling in neurons is essential for its outgrowth. XBP1 deficiency impairs neurite growth indicating the activation of IRE1α-xbp1 is essential in developing central nervous system (CNS) of mammals (Hayashi and Su, 2007; Edvardson et al., 2019). This intricate regulation of synaptic function by the UPR is evident in various neurodegenerative disorders. Phosphorylation of eIF2α plays a central role in the synaptic dysfunction and memory impairments observed in Alzheimer’s, tauopathies, and prion diseases. Reducing p-eIF2α levels or modulating its upstream kinases (e.g., PERK, GCN2, PKR) has improved synaptic plasticity and memory in animal models (Smith and Mallucci, 2016). Moreover, XBP1s enhance cognitive function by regulating BDNF and dendritic spine-associated proteins, indicating the broader implications of UPR components in neuropsychiatric and neurodegenerative disorders (Gerakis and Hetz, 2018). A study on mouse brain aging demonstrated that impaired UPR signaling begins by middle age and ablation of IRE1 expression accelerates age-associated cognitive decline (Cabral-Miranda et al., 2022).

MAM further complicates the ER stress signaling in pathological conditions. The close juxtaposition of the ER and mitochondria at MAMs enables efficient calcium ion transfer from ER to mitochondria, which is essential for mitochondrial ATP production, buffering of cytosolic calcium levels, and modulation of the intrinsic apoptotic pathway (Hetz et al., 2020; Kim et al., 2022). Dysfunction of MAM components and the resulting alterations in calcium signaling have been implicated in several neurodegenerative conditions, highlighting the critical interplay between PERK, IRE1, and MAM-resident proteins in maintaining neuronal integrity (Corazzari et al., 2017). The therapeutic intervention also includes the modulation of MAM proteins, sigma-1 receptor (Sig-1R), and IRE1α. Tan et al. (2024) demonstrated that activating the sigma-1 receptor enhanced motor function recovery, reduced apoptosis, and ameliorated neuronal ferroptosis via IRE1α.

## 4 Pharmacological intervention targeting IRE1 and PERK

Till now, we have described the involvement of IRE1/PERK in shaping inter-organellar communication and its participation in the neurodegenerative diseases. Due to their involvement in various physiological and pathophysiological processes, a careful pharmacological intervention could be one of the best strategies to combat diseases. A recent study elucidated the crosstalk between the IRE1 and PERK branches of UPR during prolonged ER stress through pharmacological inhibition. The authors demonstrated that IRE1-XBP1s signaling is crucial in sustaining PERK expression under chronic stress conditions. Treatment with various IRE1 inhibitors, including MKC8866, reduced PERK signaling during extended ER stress, suggesting that these inhibitors may also suppress PERK expression and activity. Furthermore, the study shows that IRE1’s RNase activity enhances PERK transcription through the IRE1-XBP1s axis. These findings highlight that the IRE1, PERK, and ATF6 pathways function as an interconnected signaling network, rather than as isolated, parallel branches of the UPR (Ong et al., 2024). IRE1 inhibitors can work by inhibiting both the kinase and the RNase domain or blocking either the kinase or the RNase domain individually. Thus, these inhibitors block IRE1 phosphorylation-driven signaling cues or IRE1 signaling activated by the RNase domain (e.g., RIDD and XBP1 splicing). This specific activity provides a useful strategy to target the required signaling depending on the disease.

Inhibitors like 4μ8c and STF-083010 have been found effective against cancer cell proliferation, demonstrating the potential of inhibiting pro-survival UPR functionality by blocking RIDD (Papandreou et al., 2011; Cross et al., 2012). Kinase domain-specific inhibitor of IRE1α, kinase-inhibiting RNase attenuators 6 (KIRA6), an ATP-competitive inhibitor, disrupts IRE1α oligomerization, reducing RNase activity and promoting cell survival under stress. Kinase domain-specific inhibitor of IRE1α shows therapeutic potential in conditions like retinal degeneration and hyperglycemia by protecting photoreceptors and pancreatic β cells (Cross et al., 2012; Guirao-Abad et al., 2020). With respect to multiple myeloma (MM) treatment, bortezomib (BTZ), a proteasome inhibitor, is often found to be effective for very short duration. The drug resistance tends to develop rapidly against BTZ. To overcome the limitation and in search of new therapeutics, Ri et al. used toyocamycin, an XBP1 inhibitor derived from an Actinomycete strain. Toyocamycin selectively inhibits the IRE1α-XBP1 pathway by preventing XBP1 mRNA cleavage without activating ATF6 or PERK signaling. It blocks both constitutive and ER stress-induced XBP1 mRNA splicing and induces apoptosis even in bortezomib-resistant MM cells. Additionally, toyocamycin with BTZ showed a synergistic effect over MM (Ri et al., 2012).

Dual inhibitors, such as MKC-3946 and GSK2850163, are also effective in cancer treatment by modulating kinase-driven XBP1 splicing and RNase-driven mRNA decay, offering therapeutic potential for chronic ER stress-related diseases. MKC-3946 enhances CHOP-mediated apoptosis and blocks XBP1 splicing induced by chemotherapeutic agents, making it a promising candidate for improving cancer treatment outcomes, particularly in MM. The above-described inhibitors are mainly effective against different cancers where ER stress sensors play a significant role in cell survival. Interestingly, while inhibiting IRE1 function, with these inhibitors also alter mitochondrial activity, indicating disruption of ER-mitochondrial crosstalk.

STF-083010, an inhibitor of IRE1α endonuclease activity, significantly impacts mitochondrial functions, including decreased maximal mitochondrial respiration and spare respiratory capacity. It has also been observed that it can alter mitochondrial membrane potential and increase mitochondrial sensitivity to uncouplers. Importantly, these effects are independent of inhibiting IRE1α endonuclease activity, possibly due to STF-083010’s hydrophobic nature, which allows it to accumulate in cell membranes and directly act on mitochondrial dynamics (Hatokova et al., 2023).

Another IRE1α inhibitor, 4μ8c, plays an important role in modulating ER-mitochondria crosstalk. Aβ treatment has been shown to increase the expression of MAM proteins such as IP3R, Grp75, and VDAC1, leading to increased ER-mitochondria connection and then damage of MAMs and mitochondrial dysfunction. Pre-treatment with 4μ8c rescued the length of ER-mitochondria contact sites and also restored calcium homeostasis. Similarly, APY29 significantly affects ER-mitochondria crosstalk by disrupting cellular homeostasis and inducing apoptosis in ovarian granulosa cells (SVOG). APY29 has shown to intensify reactive oxygen species (ROS) and also decrease mitochondria membrane potential significantly; both are critical indicators of mitochondrial health and function (Li et al., 2025b).

PERK inhibitors essentially block ER stress as well as ISR pathways. The ISR involves four key kinases, with PERK playing a central role. Upon ER stress (e.g., thapsigargin exposure), PERK phosphorylates eIF2α, suppressing global protein synthesis while promoting translation of adaptive genes like ATF4. This enhances mitochondrial proteostasis, increases phosphatidic acid on the outer mitochondrial membrane, inhibits the pro-fission protein DRP1, and promotes mitochondrial elongation—strengthening ER-mitochondria contact. ISRIB, an ISR inhibitor, counteracts eIF2α phosphorylation to test whether these mitochondrial changes are PERK-dependent (Baron et al., 2025).

GSK2606414 is a selective PERK inhibitor against high glucose-induced neurotoxicity in mouse neuroblastoma cells. It attenuates the UPR by downregulating ER stress markers such as GRP78, p-PERK, p-eIF2α, ATF4, and CHOP. This inhibition also reduces pro-apoptotic proteins Bax and caspase-3 while increasing anti-apoptotic Bcl-2 levels, highlighting its potential in mitigating ER stress-related neuronal damage (Gundu et al., 2022). GSK2606414 also enhances mitochondrial function by restoring levels of TFAM, NRF1, Complex I, Complex II, and ATP synthase subunit C. It also reverses the downregulation of MFN2, a key ER-mitochondria tethering protein, and reduces mitochondrial superoxide accumulation (Gundu et al., 2022).

In prion-infected mice, GSK2606414 demonstrates strong neuroprotective effects by inhibiting PERK phosphorylation, reducing p-eIF2α, ATF4, and CHOP levels, restoring global protein synthesis, and preserving synaptic proteins like SNAP25 and PSD95 (Moreno et al., 2013). However, at micromolar concentrations, GSK2606414, along with GSK2656157 and AMG44, can off-target activate the ISR via GCN2 due to increased ATP affinity at its kinase domain. This dose-dependent activation occurs without ER stress, underscoring the importance of precise dosing when using PERK inhibitors to study ISR-driven mitochondrial remodelling (Szaruga et al., 2023).

GSK2656157, another PERK inhibitor, exhibits neuroprotective effects in a subarachnoid haemorrhage (SAH) rat model by modulating ER stress and suppressing TXNIP signaling. Following SAH, TXNIP expression increases in neurons, microglia, and astrocytes, contributing to early brain injury via pro-inflammatory and pro-apoptotic pathways. Intra-cerebroventricular administration of GSK2656157 inhibits PERK autophosphorylation and downregulates ER stress markers (GRP78, p-PERK, p-eIF2α, ATF4, CHOP), reducing inflammation and apoptosis. It also restores mitochondrial function by increasing the expression of TFAM, NRF1, Complex I, Complex II, and ATP synthase subunit C (Yan et al., 2017). Like inhibitors, PERK activators are also effective therapeutics in certain neurodegenerative conditions. Studies of cellular and mouse models of tauopathy demonstrated that treatment of CCT020312 improved memory and motor function via reduction of tau phosphorylation in P301S tau mice (Bruch et al., 2017). Similarly, another PERK activator, MK28, reduced ER stress-induced apoptosis in a striatal cell line (ST*Hdh*Q111/111) while *in vivo* administration improved motor function and life span of the R6/2 HD mice (Ganz et al., 2020). Table 2 lists the IRE1/PERK inhibitors or activators, mentioning their known effect on mitochondrial function wherever applicable.

**Table 2:** Inhibitors of IRE1 and PERK.

Inhibitor name	Function	Modulate ER-mitochondria	Reference
IRE1-inhibitors			
STF-083010	Inhibits RNase activity, reduces ER stress, promotes apoptosis, and lowers tumor burden in MM and hepatocellular carcinoma	Disrupts ER-mitochondria crosstalk by altering mitochondrial respiration, membrane potential, and dynamics, independent of IRE1α inhibition	Papandreou et al. (2011), Papandreou et al. (2011), and Hatokova et al. (2023)
4μ8C	Selectively inhibits RNase activity, blocks RIDD without affecting kinase activity, reduces ER stress and inflammation	Rescues ER-mitochondria dysfunction by reducing contact site length, restoring calcium homeostasis, and mitigating Aβ-induced MAM damage and mitochondrial impairment	Cross et al. (2012)
MKC-3946	Dual inhibitor of RNase and kinase activities, blocks XBP1 splicing, enhances apoptosis, promising for MM therapy	Not known	Mimura et al. (2012)
Toyocamycin	Blocks the IRE1α-XBP1 pathway, induces apoptosis, inhibits tumor growth, and works even in bortezomib-resistant cells	Not known	Ri et al. (2012)
KIRA6	ATP-competitive inhibitor, disrupts IRE1α oligomers, enhances cell survival under stress, mitigates retinal degeneration, and hyperglycaemia	Not known	Ghosh et al. (2014)
(*R*)-2-(3,4-dichlorobenzyl)-*N*-(4-methylbenzyl)-2,7-diazaspiro(4.5)decane-7-carboxamide	Dual inhibition of kinase and RNase activities, which interacts with catalytic residues, induces conformational changes, preventing XBP1 mRNA splicing	Not known	Concha et al. (2015)
GSK2850163	Dual inhibitor of RNase and kinase domains, induces conformational changes, modulates both canonical and non-canonical activities	Not known	Concha et al. (2015)
B-I09 and D-F07	Suppress XBP1s expression, enhance cytotoxicity in MM cells, target non-canonical roles with ROS-sensitive modifications	inhibitors	Shao et al. (2020)
HM100168	Specifically inhibits RNase activity, reduces XBP1 levels, inhibits cancer cell proliferation, and overcomes resistance	Not known	Kim et al. (2024)
APY28 and APY29	APY28 reduces RNase activity, APY29 increases RNase activity, supports adaptive stress responses, and encourages apoptosis	APY29 disrupts ER-mitochondria crosstalk, elevates ROS, and significantly lowers mitochondrial membrane potential—triggering apoptosis in ovarian granulosa cells	Korennykh et al. (2011) and Li et al. (2025b)
MKC8866	Inhibition of IRE1 RNase activity Decreased levels of XBP1s transcript and its downstream targets endoplasmic reticulum DNA J domain-containing protein 4 (ERDJ4, also known as DNAJB9) and homocysteine-responsive ER protein with ubiquitin like domain 1 (HERP, also known as HERPUD1)	Not known	
PERK -inhibitors			
GSK2606414	Selectively inhibits PERK, suppresses UPR signaling, restores translation, reduces apoptosis, and improves mitochondrial function.	enhances mitochondrial function by restoring levels of TFAM, NRF1, Complex I, Complex II, and ATP synthase subunit C.	Moreno et al. (2013) and Gundu et al. (2022)
GSK2656157	ATP-competitive PERK inhibitor, blocks autophosphorylation, suppresses TXNIP, reduces inflammation, and apoptosis	restores mitochondrial function by increasing the expression of TFAM, NRF1, Complex I, Complex II, and ATP synthase subunit	Zhao et al. (2017)
ISRIB	Stabilizes eIF2B, counteracts eIF2α phosphorylation, restores translation, and reverses ISR-induced stress granule formation.	Targeting ATF4-DDIT4/TXNIP induced mitochondrial dysfunction and ferroptosis:	Baron et al. (2025)
PERK activator			
CCT020312	Enhanced the PERK/p-eIF2α/LC3-II autophagy signaling pathway in middle cerebral artery occlusion/reperfusion (MCAO/R) mice.	Not known	Li et al. (2025a)
MK-28	Reduction in ER stress-induced apoptosis in a striatal cell line (STHdhQ111/111) [193] derived from knock-in HD model mice. Improve motor function R6/2 TG mice model B6CBA-R6/2 (CAG 160).	Not known	Ganz et al. (2020)

## 5 Discussion

Over the last decade, remarkable progress has been made towards understanding UPR signaling through two ER-resident kinases, IRE1 and PERK. While their role in ER stress-mediated signals is extensively explored, reports on their involvement in other physiological processes are still growing. Beyond proteotoxic stress, these regulations establish their non-canonical functions from development to diseases. IRE1 and PERK orchestrate diverse cellular processes, including protein folding, synaptic function, autophagy, calcium balance, and mitochondrial homeostasis via MAMs. Notably, dysregulated PERK/eIF2α and IRE1/XBP1 pathways are implicated in synaptic plasticity deficits, cognitive impairment, and maladaptive neuroinflammation. Conversely, these pathways are also essential at developmental stages during neurite outgrowth, underlining their dual role in development and disease progression. Similarly, these sensors maintain mitochondrial function and calcium flux at a normal physiological state. In contrast, during stress, these sensors help cells to adapt to quick changes in energy demands by modulating mitochondrial bioenergetics. Overall, due to these signaling overlaps, pharmacological interventions are challenging. A recent study by the Bertolotti group demonstrated concentration-dependent differential activation observed between nanomolar versus micromolar amounts. Interestingly, the authors demonstrated that ATP-competitive inhibitors of PERK, GSK2656157, GSK2606414, and AMG44, inhibit PERK in the nanomolar range but surprisingly activate the ISR via GCN2 at micromolar concentrations (Szaruga et al., 2023). Additionally, PERK inhibitors (GSK series) have been found to inhibit other kinases, causing additional challenges for their application as therapeutics (Rojas-Rivera et al., 2017). Additionally, due to its crucial role in high levels of insulin production in the pancreas, continuous administration of PERK inhibition leads to pancreatic toxicity (Yu et al., 2015). PERK inhibitor delayed Purkinje cell degeneration, reduced the skeletal muscle abnormalities with improved motor performance during the symptomatic phase in a mouse model of Marinesco-Sjögren syndrome (Grande et al., 2018). In contrast, PERK activators are beneficial against tauopathies and HD (Ganz et al., 2020; Li et al., 2025a). Thus, it is important to understand the cellular physiology and the pathological condition before administration of any small molecules modulating PERK’s function.

Similarly, limitations of IRE1α inhibitors is linked with off-target effects, target cell types, and the inherent dual expression nature of IRE1 (Siwecka et al., 2021). For example, IRE1 inhibitors have been applied in different cancer treatment. In multiple myeloma (MM), IRE1 has a tumor-growth-promoting role, while in B-cell lymphoma germinal center B-cell-like subtype (GCB-DLBCL), IRE1/XBP1s activity may negatively impact tumor growth (Bujisic et al., 2017). Thus, the same inhibition might have opposite effects based on the cancer types. The scenario changes in the context of brain aging, where the overall decline of the prototypical network has been observed with age. The genetic disruption of the ER stress sensor IRE1 accelerated age-related cognitive decline. Thus, a beneficial effect was observed after XBP1s gene delivery to aged mice. Similarly, boosting IRE1 ameliorates ALS disease phenotypes in C9orf72 mice by clearing poly (GR) dipeptide repeats (Li et al., 2025b).

Besides its large spectrum of activities IRE1, the action of IRE1 inhibitors also play an important role. STF-083010, an inhibitor of endonuclease activity of IRE1α, decreases maximal mitochondrial respiration and spare respiratory capacity in SH-SY5Y cells, and impairment of mitochondrial functions is independent of its inhibitory activity on IRE1α (Hatokova et al., 2023). Thus, a fine dosage depending on target cells is crucial for a beneficial effect. Moreover, a recent study has indicated IRE1 and PERK crosstalk during chronic ER stress, where IRE1 signaling increases PERK expression, suggesting involvement of crosstalk between two sensors (Ong et al., 2024). Considering these complex scenarios, dissecting these interrelations more carefully is necessary. An enhanced understanding of these two sensors is still required for better therapeutic interventions.
